# Label-free high-throughput photoacoustic tomography of suspected circulating melanoma tumor cells in patients *in vivo*

**DOI:** 10.1117/1.JBO.25.3.036002

**Published:** 2020-03-13

**Authors:** Pengfei Hai, Yuan Qu, Yang Li, Liren Zhu, Leonid Shmuylovich, Lynn A. Cornelius, Lihong V. Wang

**Affiliations:** a Washington University in St. Louis, Department of Biomedical Engineering, St. Louis, Missouri, United States; b California Institute of Technology, Caltech Optical Imaging Laboratory, Andrew and Peggy Cherng Department of Medical Engineering, Pasadena, California, United States; c Washington University School of Medicine, Division of Dermatology, St. Louis, Missouri, United States; d California Institute of Technology, Caltech Optical Imaging Laboratory, Department of Electrical Engineering, Pasadena, California, United States

**Keywords:** photoacoustic imaging, melanoma, circulating tumor cell

## Abstract

**Significance:** Detection and characterization of circulating tumor cells (CTCs), a key determinant of metastasis, are critical for determining risk of disease progression, understanding metastatic pathways, and facilitating early clinical intervention.

**Aim:** We aim to demonstrate label-free imaging of suspected melanoma CTCs.

**Approach:** We use a linear-array-based photoacoustic tomography system (LA-PAT) to detect melanoma CTCs, quantify their contrast-to-noise ratios (CNRs), and measure their flow velocities in most of the superficial veins in humans.

**Results:** With LA-PAT, we successfully imaged suspected melanoma CTCs in patients *in vivo*, with a CNR >9. CTCs were detected in 3 of 16 patients with stage III or IV melanoma. Among the three CTC-positive patients, two had disease progression; among the 13 CTC-negative patients, 4 showed disease progression.

**Conclusions:** We suggest that LA-PAT can detect suspected melanoma CTCs in patients *in vivo* and has potential clinical applications for disease monitoring in melanoma.

## 1 Introduction

More than 90% of cancer-associated mortality is caused by metastasis,^1^ the process of cancer cells spreading from a primary tumor site to surrounding tissues and distant organs to form new tumors. During the entire metastatic process, a cancer cell executes multiple steps, including acquiring an invasive phenotype, invading the surrounding tissue, entering the local blood or lymphatic vessel (intravasation), surviving and circulating in the blood stream, exiting through vessels to distant tissues (extravasation), adapting to the new microenvironment, and growing into metastases.^2^ In this complicated metastatic cascade, the rare circulating tumor cells (CTCs) are key determinants of metastatic propensity.^3^^,^^4^ Considered as “seeds” of metastases, the presence and concentration of CTCs are closely correlated with tumor progression, metastases, and survival rates in patients.^5^ Thus, efficient detection and characterization of CTCs will make critical contributions to cancer diagnosis and staging, therapy response assessment, and residual disease evaluation after surgery.

Recent advances in biomedical techniques have enabled intensive studies of CTCs in the bloodstream,^6^ typically utilizing tumor-specific physical and biological properties to detect, capture, or isolate CTCs. However, these methods have certain limitations. Most *ex vivo* CTC detection assays, including the CTC microchip and the FDA-approved CellSearch (CELLSEARCH®), rely on epithelial markers.^7^^,^^8^ A subpopulation of CTCs could be missed in such assays because some CTCs can shield their epithelial markers or express fewer markers after transition. A new generation of microchip was developed to isolate CTC clusters based on size.^9^ However, it is constrained by the limited blood sample volume in *ex vivo* assays relative to the patient’s entire blood volume, which reduces the sensitivity of CTC detection. To improve the detection sensitivity, *in vivo* optical CTC imaging techniques have also been developed,^10^ including *in vivo* fluorescent flow cytometry,^11^ *in vivo* photoacoustic flow cytometry,^12^ and multiphoton microscopy.^13^ However, most of these techniques require labeling CTCs by transfection, and their clinical translation is impeded by low *in vivo* transfection efficiency as well as the potential toxicity of labeled CTCs. Furthermore, because of strong optical scattering in biological tissue, prior noninvasive optical CTC imaging methods rely on imaging special anatomical sites, such as the mouse ear.^12^ Sites with similar optical properties may not be readily available for human studies. Even if these techniques can be applied to detect CTCs at a favorable anatomical site, these methods may be incapable of monitoring early metastasis away from this site.^14^ Therefore, a new imaging method that can monitor metastasis using endogenous contrast over a long duration at an arbitrary anatomical location is desirable.

By ultrasonically imaging optical absorption contrast, photoacoustic tomography (PAT) achieves label-free imaging of biological tissue *in vivo* beyond the optical diffusion limit for tissue penetration and provides new opportunities for CTC imaging. The weak ultrasonic scattering in soft tissue allows PAT to access large blood vessels with high flow rates at depths, achieving a higher throughput for CTC detection than pure optical techniques. In this work, we focus on CTC detection in melanoma, the most fatal type of skin cancer, with more than 87,000 new diagnoses and 10,000 deaths each year.^15^ Melanoma has a high tendency to metastasize, after which the 5-year survival rate decreases from 98.5% to <20%. Effective detection of CTCs is critical for determining the risk of disease progression, understanding metastatic pathways, and facilitating early and effective clinical intervention. PAT is particularly suitable for melanoma CTC detection owing to the strong optical absorption of the highly expressed melanin, which serves as an intrinsic contrast.^16^

Herein, we describe the development of a PAT system based on a linear ultrasonic transducer array (LA-PAT) and present label-free high-throughput imaging of suspected melanoma CTCs, in humans *in vivo* for the first time. We initially imaged flowing melanoma tumor cells in microtubes *in vitro* to demonstrate the capability of LA-PAT. Then, we imaged 16 stage III and IV melanoma patients with LA-PAT and successfully detected suspected melanoma CTCs in three patients. The imaging results of suspected melanoma CTCs were validated with contrast-to-noise ratio (CNR) analysis among healthy volunteers, CTC-negative patients, and CTC-positive patients. The clinical relevance of suspected CTC detection was studied by follow-up monitoring of the patients imaged.

## 2 Methods

### 2.1 Determining the Optimal Excitation Wavelength for Photoacoustic Imaging of Circulating Melanoma Tumor Cells

Achieving the highest detection sensitivity of melanoma CTCs in blood requires choosing an excitation wavelength that maximizes the contrast between melanoma CTCs (the target) and blood (the background). We compared the optical absorption spectra of melanoma tumor cells (assuming an average 43% volume fraction of melanosome, as found in typical darkly pigmented skin cells),^17^^,^^18^ and red blood cells (RBCs) in venous blood (assuming 85% oxygen saturation^19^) in Fig. S1(A) in the Supplemental Materials. Based on the absorption spectra, we calculated the optical absorption coefficient ratio of melanoma tumor cells to RBCs in venous blood to select the wavelength that maximizes the contrast. Venous blood was used as the background here because veins, at shallow depths in tissue and with large blood volumes, are ideal imaging targets for detecting suspected melanoma CTCs in patients. Based on the optical absorption coefficient ratio, an excitation wavelength of 680 nm was chosen to maximize the contrast between melanoma CTCs and blood and achieve the highest detection sensitivity [Fig. S1(B) in the Supplemental Materials].

An excitation wavelength of 680 nm not only maximizes the photoacoustic signal ratio of a melanoma CTC to an RBC but also allows sufficient light penetration in biological tissue to access large blood vessels at depths. However, locating blood vessels in patients may be difficult, given the weak optical absorption of hemoglobin at 680 nm. This minor drawback was overcome using an excitation wavelength of 850 nm to locate the blood vessels prior to switching to 680 nm for melanoma CTC imaging. The 40-MHz center frequency of the ultrasonic transducer array was chosen in order to provide sufficient sensitivity to detect single melanoma CTCs while ensuring that the imaging depth was adequate to screen large blood vessels for high-throughput CTC imaging. A lower ultrasonic frequency, such as 21 MHz, can yield deeper penetration in biological tissue.^20^ However, a 21-MHz transducer array has only ∼45% CTC detection sensitivity in comparison with the 40-MHz transducer array at the same depths, because the number of RBCs in one resolution voxel is increased by a factor of ∼5. A higher ultrasonic frequency, however, such as 50 MHz, can achieve an even higher melanoma CTC detection sensitivity, but suffers from stronger acoustic attenuation and the inability to reach the deep target vessels for high-throughput melanoma CTC imaging. Therefore, an illumination wavelength of 680 nm and a detection center frequency of 40 MHz represent the most efficacious combination to enable label-free high-throughput melanoma CTC imaging in patients *in vivo*.

### 2.2 Linear-Array-Based Photoacoustic Tomography System for Imaging Circulating Melanoma Tumor Cells

To achieve label-free high-throughput imaging of melanoma CTCs, we upgraded a PAT system (Visualsonics Inc., Vevo LAZR) based on an LA-PAT [Fig. S5 in the Supplemental Materials). After careful analysis, an excitation wavelength of 680 nm was chosen to maximize the contrast between melanoma CTCs and blood [Fig. S1(B) in the Supplemental Materials] and another excitation wavelength of 850 nm was chosen to locate blood vessels in melanoma patients. To provide excitation wavelengths of 680 and 850 nm, a tunable optical parametric oscillator laser (680 to 970 nm, 20-Hz pulse repetition rate) was used, and the excitation wavelength could be manually switched. To ensure the safety of patients, the laser beam was coupled into an optical fiber bundle. The optical fiber bundle bifurcated, and laser beams coming out of the optical fiber bundles illuminated the imaging area at a 30-deg angle of incidence to the imaging plane. The fluence on the tissue surface during the experiments was $∼11\;\mathrm{mJ}/{\mathrm{cm}}^{2}$, well within the $\text{20 }\mathrm{mJ}/{\mathrm{cm}}^{2}$ safety limit set by the American National Standards Institute.^21^ The generated photoacoustic waves were detected by a linear array ultrasonic transducer (Visualsonics, Inc., MS550D, 40-MHz center frequency, 33-MHz bandwidth, and 256 elements). Four-to-one multiplexing was used in image acquisition because there were 256 elements in the linear array transducer and 64 channels in the data acquisition unit. In detail, for each laser pulse, the generated photoacoustic signals were captured sequentially by a quarter segment of the linear array (i.e., elements 1 to 64, 65 to 128, 129 to 192, and 193 to 256). A two-dimensional photoacoustic image was reconstructed with the universal backprojection algorithm after the data were acquired from all four quarter segments. The linear array ultrasonic transducer was connected to the imaging platform, where the reconstructed photoacoustic images were displayed. The imaging frame rate was 5  frames/s, determined jointly by the 20-Hz laser repetition rate and four-to-one multiplexing in image acquisition. The frame rate could be adjusted by utilizing fewer elements in the linear array transducer, at the expense of a smaller imaging field of view (FoV).

### 2.3 Detection Sensitivity of the LA-PAT System

The detection sensitivity of LA-PAT ultimately depends on the CNR, which allows visualizing a melanoma CTC against the background of RBCs. The CNR can be expressed by the following equation when the fluctuation of the number of RBCs in each resolution voxel is the dominant noise (Note S1 in the Supplemental Materials):

$$\mathrm{CNR}=\frac{\frac{{\mathrm{PA}}_{\mathrm{CTC}}}{{\mathrm{PA}}_{\mathrm{RBC}}}}{\sqrt{{\left[\mathrm{RBC}\right]}_{\mathrm{wo}}}}.$$ (1)

Here, ${\mathrm{PA}}_{\mathrm{CTC}}$ and ${\mathrm{PA}}_{\mathrm{RBC}}$ stand for the average photoacoustic signals of a melanoma CTC and an RBC, and ${\left[\mathrm{RBC}\right]}_{\mathrm{wo}}$ represents the average number of RBCs in a resolution voxel without circulating cells containing melanin. The photoacoustic signal ratio of a melanoma CTC and an RBC, at the same optical fluence, is determined by the absorption coefficient ratio and the cell volume ratio. The absorption coefficient ratio can be maximized by optimizing the illumination wavelength. The average number of RBCs in each resolution voxel is determined by the RBC number density and the resolution voxel size, which is affected by the ultrasonic transducer frequency.

### 2.4 Spatial Resolutions of the LA-PAT System

The spatial resolutions of the LA-PAT system were quantified by imaging a carbon fiber with a diameter of 6  μm. First, the carbon fiber was placed perpendicular to the imaging plane, and a cross section image of the carbon fiber was acquired by LA-PAT [Fig. S6(A) in the Supplemental Materials]. The photoacoustic amplitude profile of the carbon fiber along the axial direction was fitted to a Gaussian function, and the axial resolution of the LA-PAT system, measured as the full-width at half-maximum, was quantified to be 43  μm [Fig. S6(B) in the Supplemental Materials]. The LA-PAT system was then scanned along the elevational direction with a step size of 20  μm. To quantify the lateral resolution, a maximum amplitude projection (MAP) image was obtained along the axial direction of the carbon fiber [Fig. S6(C) in the Supplemental Materials]. Similarly, the photoacoustic amplitude profile of the carbon fiber along the lateral direction was fitted to a Gaussian function, and the lateral resolution of the LA-PAT system was quantified to be 94  μm [Fig. S6(D) in the Supplemental Materials]. To quantify the elevation resolution, the carbon fiber was placed along the lateral direction of the LA-PAT system. The LA-PAT system was then scanned along the elevational direction with a step size of 20 μm, and an MAP image along the axial direction of the carbon fiber was obtained [Fig. S6(E) in the Supplemental Materials]. The photoacoustic amplitude profile of the carbon fiber along the elevational direction was fitted to a Gaussian function, and the elevational resolution of the LA-PAT system was quantified to be 633  μm [Fig. S6(F) in the Supplemental Materials].

### 2.5 Cell Culture

A B16 mouse melanoma cell line was obtained from the Tissue Culture and Support Center at the Washington University School of Medicine. The B16 cells were cultured in Dulbecco’s modified Eagle’s medium (Invitrogen), supplemented with 10% fetal bovine serum (Gibco), at 37°C in 5% ${\mathrm{CO}}_{2}$. At 75% to 80% confluence, cells were harvested with 0.25% trypsin-EDTA solution (Gibco).

### 2.6 Phantom Preparation

To mimic blood vessels of different diameters, silicone microtubes (Dow Corning) with inner diameters of 0.3, 0.5, 0.64, 0.76, 1, 1.5, 2, 2.64, and 3.35 mm were perfused with bovine blood (905, Quad-Five). The microtubes were embedded at different depths in tissue-mimicking gelatin phantoms (10% gelatin, G2500, Sigma-Aldrich, Inc.). To achieve optical scattering similar to that in biological tissue, 1% intralipid was added to the gelatin phantoms. The acoustic properties of tissue-mimicking phantoms have been reported.^22^ Melanoma tumor cells suspended in bovine blood were pumped through the microtubes with a syringe. A syringe pump (BSP-99M, Braintree Scientific) controlled the blood flow speed.

### 2.7 Detection Efficiency Calculation

To quantify the melanoma tumor cell detection efficiency of LA-PAT, ∼1000 single melanoma tumor cells were suspended in 10-mL bovine blood and pumped through a microtube with a syringe. The melanoma tumor cells and blood were collected at the other end of the microtube. After ∼50 melanoma tumor cells had been captured by LA-PAT, the collected melanoma tumor cells and blood mixture were examined with an optical microscope to count the number of melanoma tumor cells pumped through the microtube. The detection efficiency E is defined as

$$E=\frac{n}{N}.$$ (2)

Here, n stands for the number of melanoma tumor cells captured by LA-PAT, and N stands for the number of melanoma tumor cells counted by optical microscopy.

### 2.8 Imaging Procedure for Melanoma Patients

This protocol was approved by the Institutional Review Board of Washington University in St. Louis (IRB #201410125), and the study was part of a larger clinical trial (Clinical registration: Clinicaltrials.gov NCT02613325). In this trial, the primary purpose was to use a PAT system to determine melanoma depth in the skin, and the secondary purpose was to assess the feasibility and functionality of a PAT system in the detection of suspected CTCs. A total of 24 subjects were enrolled in this trial, and of the 16 patients described in the current study (Table S2 in the Supplemental Materials), three patients met inclusion criteria for both primary and secondary outcomes. Results for the primary outcome have been previously published.^23^

For this study, after informed consent was obtained, the patients were seated in a large chair or were lain on a portable patient bed. Depending on the location of the melanoma or known metastases, either the cephalic veins or the saphenous veins were imaged—the vessel proximal to the melanoma tumor site was chosen for imaging. First, an excitation wavelength of 850 nm was used to locate the melanoma and then its proximate vein (Fig. S7 in the Supplemental Materials). Second, we moved the linear array ultrasonic transducer downstream until we found the most superficial section of the vein in the patient’s forearm or leg. Finally, we kept the linear array ultrasonic transducer at this location and switched to 680 nm to detect melanoma CTCs. Each patient was usually imaged for 20 min, and during the entire imaging session, laser safety glasses with an optical density >5 at 680 and 850 nm were worn to ensure the safety of the patient and operator.

### 2.9 Flow Speed of Suspected Melanoma Tumor Cells

To make robust measurements of the flow speed of suspected melanoma tumor cells, we converted their motion images to the space–time domain. The time traces of each pixel along the yellow dashed line in Figs. S2(A) and S2(B) in the Supplemental Materials were extracted, and the signals were shown as an image in the space–time domain (Fig. S2 in the Supplemental Materials). After the data were fitted to a linear function, the slope of the linear function in the space–time domain was used to calculate the speed of the melanoma CTCs.

## 3 Results

### 3.1 LA-PAT of Melanoma Tumor Cells in Phantoms

**Fig. 1:**
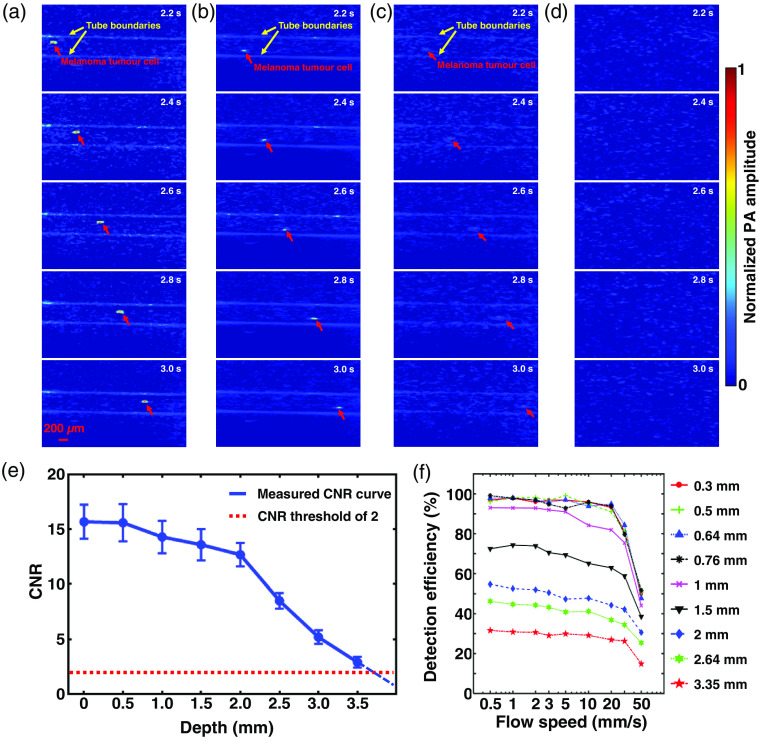
LA-PAT of single melanoma tumor cells in phantom. (a) Photoacoustic snapshots of a single melanoma tumor cell in a transparent phantom with a CNR of 16.4. The yellow arrows show the microtube boundaries. The red arrows highlight the circulating single melanoma tumor cell [Video 1(a)]. (b) Photoacoustic snapshots of a single melanoma tumor cell 1.5 mm deep in a scattering phantom with a CNR of 14.2 [Video 1(b)]. (c) Photoacoustic snapshots of a single melanoma tumor cell 3 mm deep in a scattering phantom with a CNR of 4.3 [Video 1(c)]. (d) Photoacoustic snapshots of the scattering phantom at a depth of 4 mm. No melanoma tumor cells are visible. (e) CNRs of the melanoma tumor cells in photoacoustic images degrade with increasing imaging depth. With a CNR threshold of 2, the maximum depth at which a melanoma tumor cell could be detected was ∼3.5  mm. (f) The melanoma tumor cell detection efficiency of LA-PAT decreases with increasing blood flow speed and vessel diameter.

**Fig. 2:**
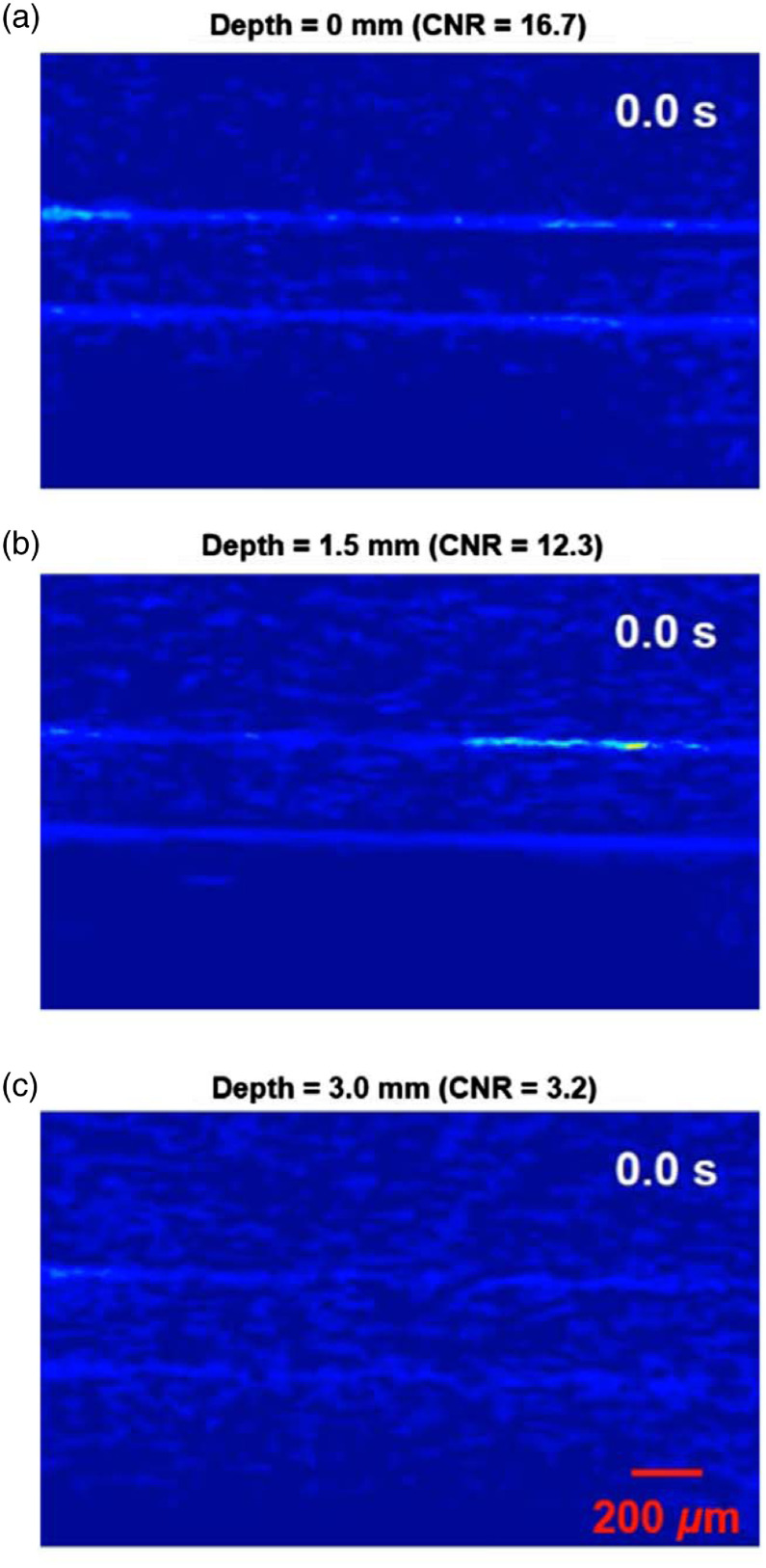
LA-PAT of flowing single melanoma tumor cells at different depth in phantoms (Video 1, MP4, 1.1 KB [URL: https://doi.org/10.1117/1.JBO.25.3.036002.1]).

To initially demonstrate the capability of LA-PAT, we first applied it to image melanoma tumor cells in tissue-mimicking phantoms. An excitation wavelength of 680 nm was used, which maximized the contrast between melanoma tumor cells and blood (Fig. S1 in the Supplemental Materials). Due to the strong optical absorption of melanin at 680 nm, the flowing melanoma tumor cells emitted much stronger photoacoustic signals than the blood in the background and were detected by LA-PAT [Figs. 1(a) and 2(a)].

The ability to detect and visualize melanoma tumor cells in the bloodstream depends on the CNR (Note S1 in the Supplemental Materials). To determine the maximum depth at which melanoma tumor cells could be detected by LA-PAT, we studied how the imaging depth affected the CNR. Microtubes mimicking blood vessels were embedded in scattering gelatin phantoms at different depths, and melanoma tumor cells suspended in blood were pumped through the microtubes. We used LA-PAT to image the flowing melanoma tumor cells [Figs. 1(a)–1(d), 2(b), and 2(c)] and calculated the CNR at each depth [Fig. 1(e)]. The CNR decreases with the increasing imaging depth because both the optical fluence and the acoustic signal attenuate. With a CNR threshold of 2, the maximum depth at which a melanoma tumor cell could be detected by LA-PAT with a confidence level of 94.5% was ∼3.5  mm. This depth is adequate for imaging most superficial veins in humans.

We also studied the melanoma tumor cell detection efficiency of LA-PAT. Despite high detection sensitivity, there are two situations where LA-PAT could miss a melanoma tumor cell. First, a melanoma tumor cell could flow outside of FoV, as when the diameter of the blood vessel is larger than the slice thickness of the ultrasonic detection, i.e., the elevational resolution. Second, a melanoma tumor cell could flow too quickly through the FoV. To quantify the detection efficiency, we imaged melanoma tumor cells at different flow speeds in microtubes with different diameters and calculated the detection efficiencies for different parameter combinations [Fig. 1(f)]. Detection efficiency decreases with increasing blood flow speed and vessel diameter. These detection efficiency curves may serve as a reference for estimating the total number of melanoma CTCs from the number detected by LA-PAT.

### 3.2 LA-PAT of a Single Suspected Melanoma CTC and a Suspected Melanoma CTC Cluster in Patient 1

**Fig. 3:**
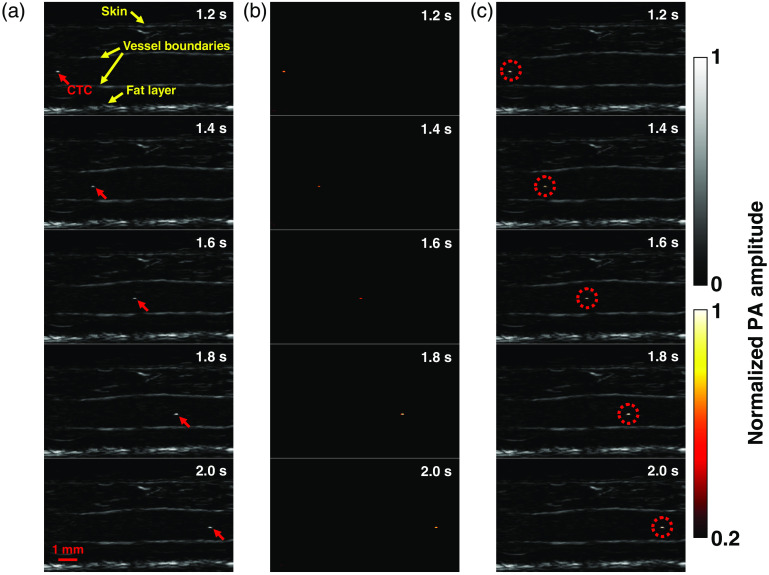
LA-PAT of a single suspected melanoma CTC in patient 1. (a) Photoacoustic snapshots of the melanoma CTC in the patient. The yellow arrows indicate structures, including the skin, vessel boundaries, and subcutaneous fat layer. The red arrows highlight the melanoma CTC. (b) Differential photoacoustic images showing only the melanoma CTC. (c) Differential photoacoustic images (b) superimposed on structural photoacoustic images (a), highlighting the melanoma CTC [Video 2(a)].

The first positive patient (M3) had stage IIIB recurrent metastatic melanoma with multiple in-transit metastases of the right lower extremity (Table S1 in the Supplemental Materials). The small saphenous vein of the right leg, with a diameter of ∼1.7  mm, was imaged. The vein measured ∼3.1  mm from the skin surface to the deep vessel boundary. In the photoacoustic images, structures including skin, vessel boundaries, and subcutaneous fat were clearly resolved [Fig. 3(a)].

**Fig. 4:**
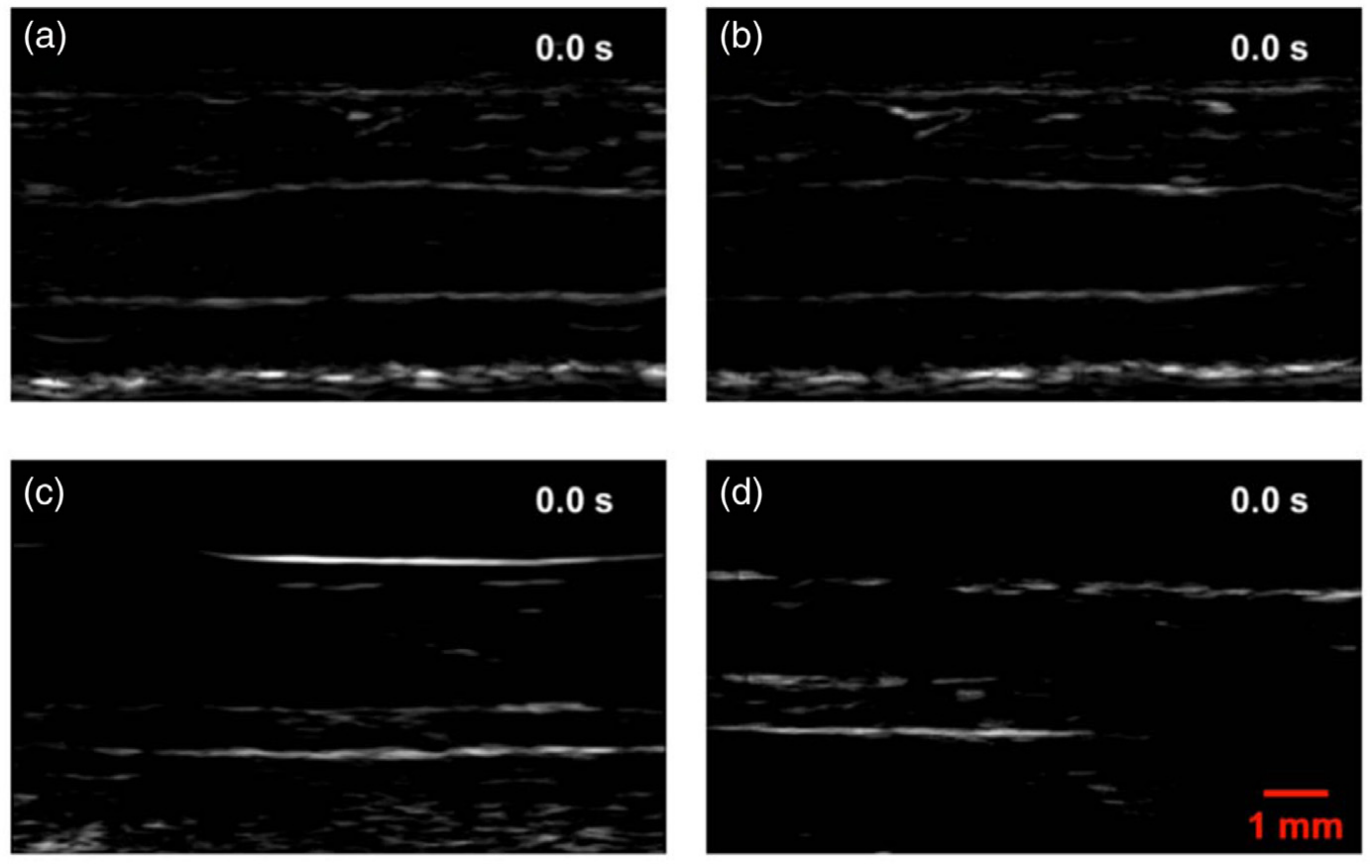
LA-PAT of melanoma CTCs in patients (Video 2, MP4, 1.5 KB [URL: https://doi.org/10.1117/1.JBO.25.3.036002.2]).

In one 7-min imaging session, LA-PAT detected a single melanoma CTC in this patient. The single cell, with a CNR of ∼12.6, was captured at five instants during its passage through the entire FoV [Fig. 3(a)]. Differential photoacoustic images were obtained [Fig. 3(b)], then superimposed on the structural images to better illustrate how the cell traversed the entire FoV [Figs. 3(c) and 4(a)]. Pixelwise subtraction of one frame from the next frame generated a differential image. A global threshold was applied to differential images, with a threshold level set at two times the noise level, estimated as the standard deviation of the background signal outside the imaged region. The flow speed of the CTC was computed to be ∼10.3  mm/s by analyzing its movement in the space–time domain (Fig. S2 in the Supplemental Materials). Although the flow speed does not exactly match the flow speed of blood, it is a close approximation.

**Fig. 5:**
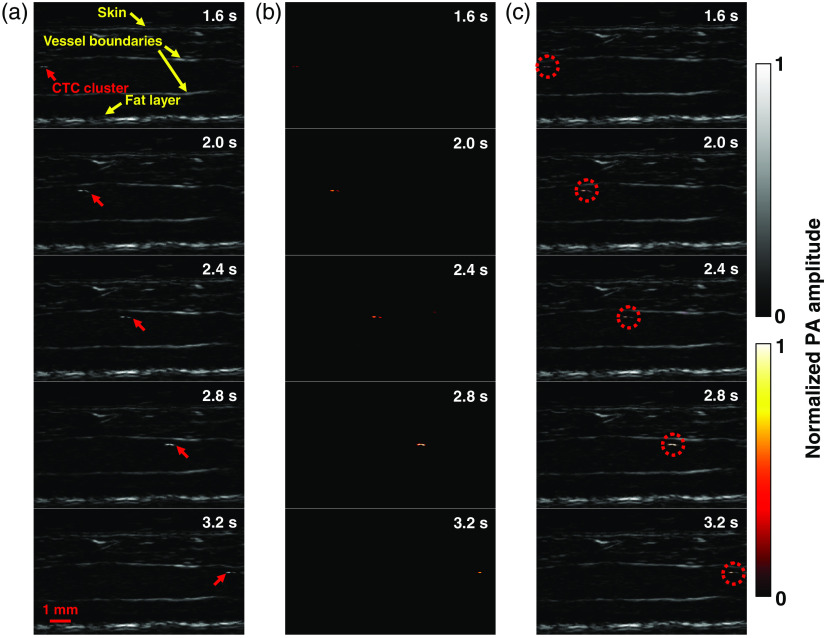
LA-PAT of a suspected melanoma CTC cluster in patient 1. (a) Photoacoustic snapshots of the melanoma CTC cluster in the patient. The yellow arrows indicate structures, including the skin, vessel boundaries, and subcutaneous fat layer. The red arrows highlight the melanoma CTC cluster. (b) Differential photoacoustic images showing only the melanoma CTC cluster. (c) Differential photoacoustic images (b) superimposed on structural photoacoustic images (a), highlighting the melanoma CTC cluster [Video 2(b)].

In another 7-min imaging session with this patient, LA-PAT detected a CTC cluster. The cluster, with a CNR of ∼11.6, was captured at nine instants as it traversed the entire imaging FoV [Fig. 5(a)]. It was captured more often than the single cell because of its lower flow speed. As before, differential photoacoustic images were obtained [Fig. 5(b)] and superimposed on the structural images to illustrate the passage through the entire FoV [Figs. 4(b) and 5(c)]. Applying the same method as for the single cell, the flow speed of the cluster was computed to be ∼5.4  mm/s (Fig. S2 in the Supplemental Materials). A possible explanation for the lower flow speed of the cluster compared to the single CTC is that the cluster was closer to the vessel boundary, while the single cell was situated closer to the vessel centerline. Assuming a parabolic flow, the flow speed near the centerline would be greater than that near the vessel boundary.

### 3.3 LA-PAT of a Single Suspected Melanoma CTC in Patient 2

**Fig. 6:**
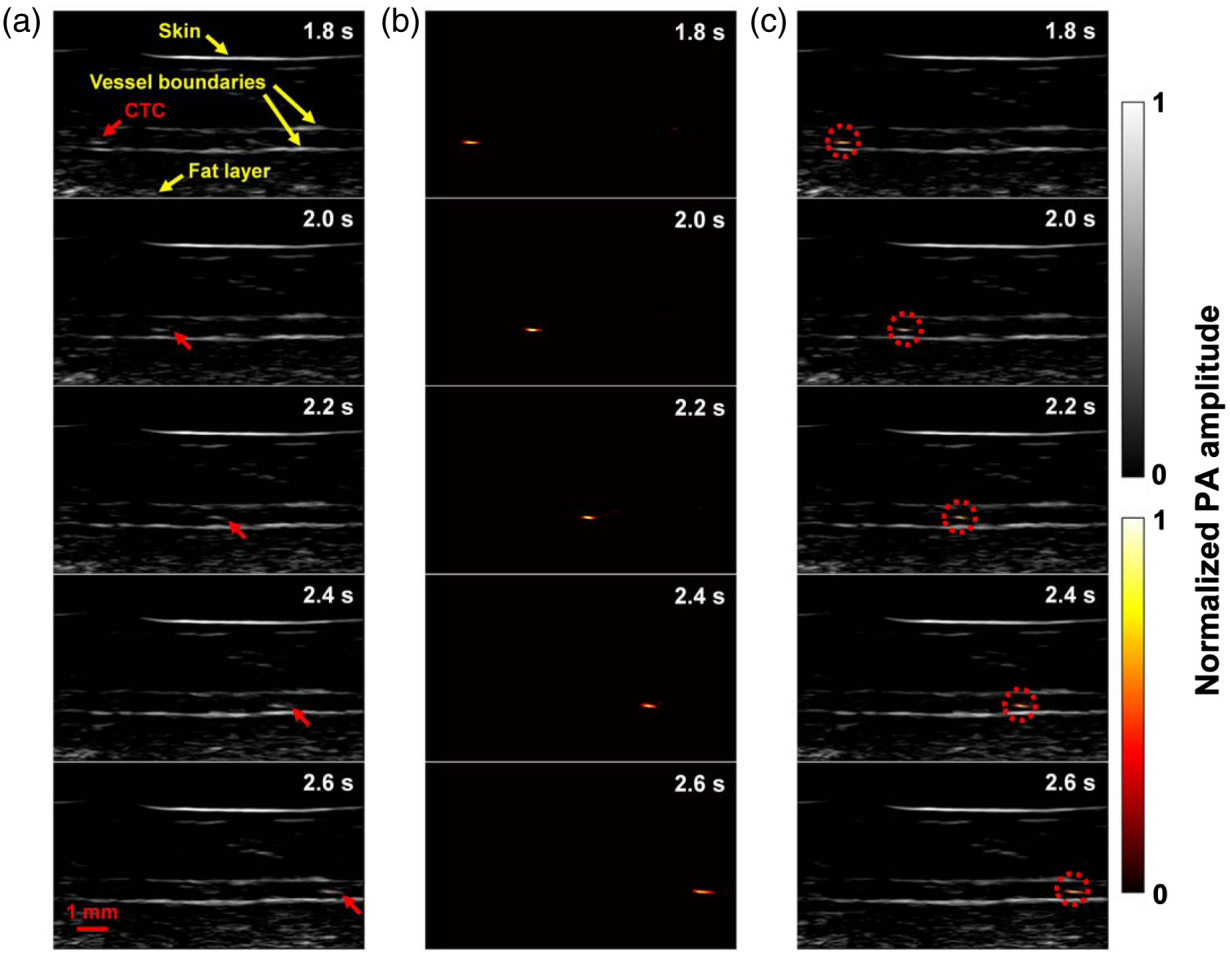
LA-PAT of a single suspected melanoma CTC in patient 2. (a) Photoacoustic snapshots of the melanoma CTC in the patient. The yellow arrows indicate structures, including the skin, vessel boundaries, and subcutaneous fat layer. The red arrows highlight the melanoma CTC. (b) Differential photoacoustic images showing only the melanoma CTC. (c) Differential photoacoustic images (b) superimposed on structural photoacoustic images (a), highlighting the melanoma CTC [Video 2(c)].

The second positive patient (M11) had stage IV metastatic melanoma (Table S1 in the Supplemental Materials). The cephalic vein of the right forearm, with a diameter of ∼10  mm, was imaged. The vein measured ∼3.2  mm from the skin surface to the deep vessel boundary [Fig. 6(a)].

In one 7-min imaging session, LA-PAT detected a single melanoma CTC in this patient, with a CNR of ∼9.4. The single cell was captured at five instants during its traverse of the entire FoV [Fig. 6(a)]. As before, differential photoacoustic images were obtained [Fig. 6(b)] and then superimposed on the structural images to illustrate how the melanoma CTC traversed the entire FoV [Figs. 4(c) and 6(c)]. Per previous calculations, by converting the movement of the single cell to the space–time domain, the flow speed of the melanoma CTC was estimated to be ∼9.6  mm/s.

### 3.4 LA-PAT of a Suspected Melanoma CTC Cluster in Patient 3

**Fig. 7:**
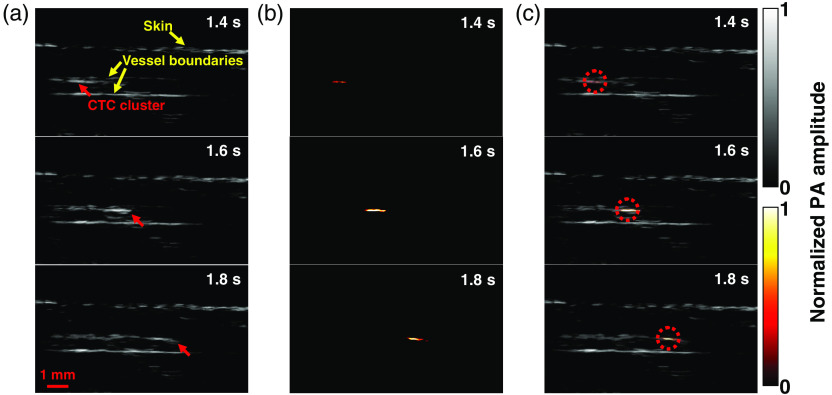
LA-PAT of a suspected melanoma CTC cluster in patient 3. (a) Photoacoustic snapshots of the melanoma CTC cluster in the patient. The yellow arrows indicate structures, including the skin and vessel boundaries. The red arrows highlight the melanoma CTC cluster. (b) Differential photoacoustic images showing only the melanoma CTC cluster. (c) Differential photoacoustic images (b) superimposed on structural photoacoustic images (a), highlighting the melanoma CTC cluster [Video 2(d)].

The third positive patient (M14) had stage IIIC metastatic melanoma with both in transit metastasis and nodal disease (Table S1 in the Supplemental Materials). The cephalic vein of the patient’s right forearm, with a diameter of ∼10  mm, was imaged. The vein measured ∼2.2  mm from the skin surface to the deep vessel boundary. In the photoacoustic images, structures, including the skin and vessel boundaries, were clearly resolved [Fig. 7(a)].

In one 7-min imaging session, LA-PAT detected a melanoma CTC cluster in this patient. The CNR of the melanoma CTC cluster was ∼12.5. The melanoma CTC cluster was captured at three instants as it traversed the entire FoV [Fig. 7(a)]. Again, to highlight the circulating melanoma CTC, differential photoacoustic images were obtained [Fig. 7(b)]. The differential photoacoustic images were then superimposed on the structural images to better illustrate how the melanoma CTC cluster traversed the entire FoV [Figs. 4(d) and 7(c)]. The flow speed of the melanoma CTC cluster was estimated to be ∼8.6  mm/s.

### 3.5 CNR Analysis of Suspected Melanoma CTCs

To confirm that the high-CNR circulating entities detected by LA-PAT were attributed to cells containing a large amount of melanin, we performed a thorough CNR analysis in tissue phantoms and patients. In phantoms, blood without melanoma tumor cells was pumped through the microtube at a constant speed and imaged by LA-PAT. Considering the spatial resolution of LA-PAT, we segmented the microtube into multiple regions and defined each region as an entity. The location of one entity in the next frame was calculated from the flow speed of blood and the frame rate of LA-PAT. As expected, no flowing entities with a CNR averaged over sequential frames above 2.0 were detected in the photoacoustic images [Fig. S3(A) in the Supplemental Materials]. We then searched within the microtube to find the static pixel with the highest CNR, referred as the peak CNR. Next, pigmented B16 murine melanoma cells were suspended in blood and pumped through the microtube. LA-PAT detected high-CNR flowing entities with CNRs greater than 2.0 in the photoacoustic images [Fig. S3(B) in the Supplemental Materials]. The CNRs of the high-CNR entities with melanoma tumor cells were higher than the peak CNR of blood by a factor of ∼3.5 [Fig. S3(C) in the Supplemental Materials]. Because the added melanoma tumor cells were the only difference from the control experiment, the high-CNR flowing entities were identified as melanoma tumor cells. At an excitation wavelength of 680 nm, only a melanin-containing cell that is much more absorbing than an RBC in blood (Fig. S1 in the Supplemental Materials, and Sec. 2) could generate such strong photoacoustic signals (Note S1 in the Supplemental Materials). In addition, the average CNR of the flowing entities with melanoma cells was ∼14.7, close to the theoretically estimated value of 15 (Note S1 in the Supplemental Materials).

To further validate the identification of the high-CNR circulating entities *in vivo* as cells containing melanin, we similarly analyzed the CNRs in patients, with several control groups. First, we calculated the average CNR of each circulating entity in the photoacoustic images [Fig. S4(A) in the Supplemental Materials]. For the initial control, we compared the CNRs of the circulating entities with the peak background CNRs in the blood vessels of patients in whom high-CNR circulating entities were detected, referred to as CTC-positive patients. In frames without high-CNR circulating entities, we scanned the entire area within the blood vessel and found the pixel with the highest CNR [Fig. S4(B) in the Supplemental Materials]. In the blood vessels of CTC-positive patients, the CNRs of the high-CNR circulating entities were higher than the peak background CNRs by a factor of ∼2.7 [Fig. S4(E) in the Supplemental Materials]. For the second control, we compared the CNRs of the high-CNR circulating entities with the peak CNRs in the blood vessels of patients in whom no high-CNR circulating entities were detected, referred to as CTC-negative patients. Similarly, we scanned the entire area within the blood vessel and found the pixel with the highest CNR [Fig. S4(C) in the Supplemental Materials]. The CNRs of the high-CNR circulating entities were also higher than the peak background CNRs in the blood vessels of CTC-negative patients, by a factor of ∼2.8 [Fig. S4(E) in the Supplemental Materials]. For the third control, we compared the CNRs of the high-CNR circulating entities with the peak background CNRs in the blood vessels of healthy volunteers [Fig. S4(D) in the Supplemental Materials]. Consistent with the first two controls, CNRs of the high-CNR circulating entities in melanoma patients were higher than the peak CNRs measured in blood vessels of healthy volunteers, by a factor of ∼2.4 [Fig. S4(E) in the Supplemental Materials]. Taken together, this analysis supports the assertion that the circulating entities with high photoacoustic signals detected by LA-PAT in the three patients correspond to circulating cells containing melanin.

### 3.6 Estimation of the Total Number of CTCs and the Clinical Relevance of CTC Imaging in Patients

**Fig. 8:**
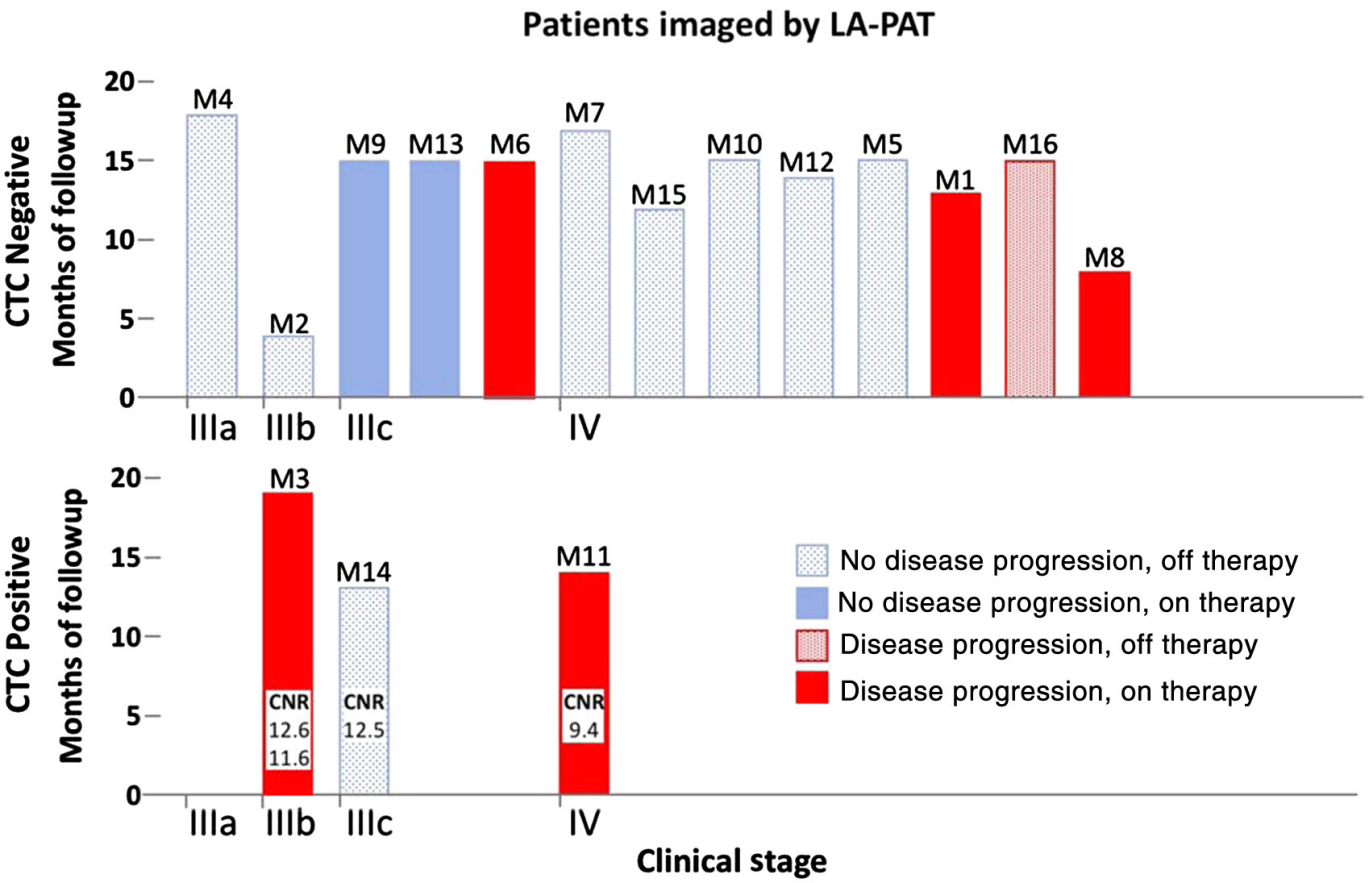
Follow-up of melanoma patients after being imaged by LA-PAT. Among three patients with detected suspected CTCs, two demonstrated disease progression on therapy, while one had no disease progression off therapy. Among 13 patients without CTCs detected, 4 demonstrated disease progression. See text for further details.

To show the potential of LA-PAT melanoma CTC imaging for analysis of metastasis, we then monitored their clinical courses for several months following photoacoustic imaging. Based on the detected numbers of suspected melanoma CTCs and the effective blood volumes screened by LA-PAT, our results were consistent with the numbers reported previously using *ex vivo* CTC detection methods^24^^,^^25^ (Table S1 in the Supplemental Materials). We wanted to determine whether the detection of suspected CTCs was predictive of patient response to treatment and/or disease progression.^26^ The data were deidentified, the results were not shared with patients or their treating physicians, and the results did not affect any clinical treatment decisions. In our study, two of the three CTC-positive patients (M3 and M11) had disease progression despite being on systemic therapy, and patient M11 ultimately died from his disease. One CTC-positive patient (M12) demonstrated no disease progression off therapy. This case may not be associated with a false-positive test result because the presence of CTCs may not always lead to disease progression. In contrast, only 4 of 13 CTC-negative patients had disease progression that may indicate false-negative results (Fig. 8).

## 4 Discussion

PAT, which is based on ultrasonically imaging optical absorption, is inherently suitable for melanoma imaging and offers four advantages. (1) PAT has a 100% relative sensitivity to optical absorption (i.e., a given small percentage change in the optical absorption coefficient yields the same percentage change in the photoacoustic amplitude), thus allowing high-sensitivity detection of strongly absorbing targets;^27^ (2) most melanoma tumor cells express high-concentration melanin, which has much stronger optical absorption than hemoglobin in the red to near-infrared spectral range and serves as an intrinsic contrast for high-sensitivity photoacoustic detection of melanoma CTCs in the bloodstream; (3) taking advantage of the ultrasonic transparency of biological tissue, PAT achieves high resolution at depths, enabling assessment of large blood vessels for high-throughput screening of melanoma CTCs; (4) LA-PAT, the linear-array-based implementation of PAT, optimally aligns the FoV with the blood vessel, which maximizes the blood imaging efficiency (i.e., the ratio of the volume of blood examined to the entire tissue volume imaged by LA-PAT) and increases the likelihood of melanoma CTCs detection in patients. Capitalizing on these advantages, we successfully achieved label-free high-throughput imaging of suspected melanoma CTCs in patients *in vivo*.

Melanoma CTC detection has been studied with multiple *ex vivo* modalities that utilize melanocyte-specific proteins and RNA sequences thought to be upregulated in melanoma cells. These modalities have been studied in patients with stage I through IV melanoma, and there is significant variation in the percentage of patients found to have CTCs and the number of CTCs detected, with estimates ranging from 13% to 80% of melanoma patients having detected CTCs.^24^ Some of the higher estimates may be the result of repeat sampling of the same patient over a study’s duration, as well as inclusion of multiple melanocyte markers. Some of the lower estimates may reflect the result of inclusion of Stage I and II patients.

Given that our study was limited to stages III and IV patients, a relevant comparable study is by Khoja et al., who reported detection of CTCs in 40% of 101 patients with stage IV melanoma.^28^ These patients were examined for the presence of CTCs multiple times during the study, and a melanoma-specific commercially available *ex vivo* CellSearch system utilizing three markers (MelCAM, melanoma-associated chondroitin sulphate proteoglycan, and CD45) was used. The number of cells detected in a 7.5-ml sample of blood for the patients with detected CTCs was 0.133  CTCs/ml for 35% of patients, 0.267  CTCs/ml for 12.5% of patients, and ≥0.4  CTCs/ml for 52.5% of patients.

We detected CTCs in 20% of stage III and IV patients. The lower rate of CTC detection compared to the work of Khoja et al. may be due in part to our utilization of only a single marker (melanin) rather than three separate markers, as well as the fact that our patients were measured at only one clinical timepoint instead of on multiple repeat visits. In our study, we detected both single CTCs and CTC clusters, and we estimate that a cluster of CTCs likely represents two to four CTC cells, based on the sizes of the clusters in the images. Using this estimate along with the effective sampled blood volume per patient suggests that ∼0.06 to 0.11  CTCs/ml were detected in patent M3, 0.07  CTCs/ml were detected in patient M11, and 0.15 to −0.30  CTCs/ml were detected in patient M14. These estimates are in overall agreement with the CTC per unit volume reported by Khoja et al.^28^

Recently, another group also reported *in vivo* melanoma detection using a photoacoustic technique,^29^ with a 96% detection rate and a 94% specificity. The duration of their examinations was longer than 1 h, in comparison with our 20-min exams. The reported number density of CTCs^29^ was consistent with the numbers that we measured from the three CTC-positive patients. Because both studies were not randomized and had a limited population size, the conclusions drawn from the two studies must be further examined in large-scale, randomized, and blinded studies.

It is worth noting that the efficacy of LA-PAT in CTC imaging depends on patient factors. First, skin pigmentation influences LA-PAT imaging. All 16 patients in the current work were Caucasians with Fitzpatrick type I-II skin. Indeed, previous studies have found that darkly pigmented skin absorbs about 30% to 40% more laser energy than lightly pigmented skin.^30^ This increased absorption would result in a simulation-predicted decrease in the maximum imaging depth of ∼2.5  mm.^31^ This depth is not a significant limitation because the epidermis can vary between 0.05 and 1.5 mm in humans, and therefore dermal blood vessels would still be accessible to LA-PAT in darker pigmented skin. Furthermore, the risk of melanoma is higher in lighter skin types than darker skin types,^32^ suggesting that LA-PAT would be particularly suited for application to most patients with malignant melanoma.

Second, the types and amounts of pigment produced in melanoma cells depend on genotypes. For example, amelanotic melanoma, a rare clinical type of melanoma that comprises less than ∼5% of the total melanoma cases,^33^^–^^36^ produces only a small amount of brown melanin (eumelanin). Fortunately, amelanotic melanoma is a rare subtype, so its potential detection limitation does not significantly limit the broader utility of LA-PAT.

While both the presence of melanin and background pigmentation present potential variables that may make CTC detection more difficult, a further question is determining whether the LA-PAT signal is indeed specific for circulating melanoma tumor cells. Our results clearly demonstrate that LA-PAT is capable of detecting flowing melanoma tumor cells in phantom experiments under conditions that are physiologically relevant to human patients. However, the three patients with detected CTC events may have had detected signals due to benign circulating melanocytes or melanin-laden macrophages instead of circulating melanoma tumor cells. Several studies have provided insight into the possible mischaracterization of benign circulating melanocytes as melanoma cells.^33^^–^^35^ De Giorgi et al. reported the first case of benign nevus cells being detected in peripheral blood samples from a patient with a benign congenital nevus.^33^ Though this case demonstrates that a patient without melanoma can have benign circulating melanocytes, other work has shown that patients with melanoma are more likely to have nevus cells found in lymph nodes,^34^^,^^35^ suggesting that patients with melanoma may be more likely to have nevus cells that acquire the ability to migrate outside of the primary cutaneous neoplasm. Nonetheless, our findings represent an important and nontrivial “proof of concept”—that melanin-containing cells can be imaged *in vivo* using LA-PAT, and that this capability has potential clinical relevance.

To confirm clinical relevance, however, larger, controlled studies are necessary. Future studies should include imaging CTCs in age-matched nonmelanoma controls and patients with multiple nevi, as well additional imaging of stages I to IV melanoma patients. Importantly, *ex vivo* validation using existing verified platforms for circulating tumor DNA (ctDNA) or other enriching platforms using tumor markers would be important in verifying that LA-PAT-determined CTCs are indeed CTCs and not benign melanin-containing cells. Furthermore, the presence of circulating melanin-containing cells in patients with late stage metastatic melanoma may be a clinically relevant finding, regardless of whether these cells are tumor cells or not.

We also propose that the functionality of LA-PAT can be further improved. Detection sensitivity can be improved by spectrally unmixing melanoma CTCs and RBCs using two or more illumination wavelengths.^37^ Blood screening throughput can be enhanced by targeting larger blood vessels and optimizing the imaging frame rate to better match the blood flow speed. Moreover, by applying a second laser pulse with higher energy immediately after identifying a melanoma CTC, selective laser fragmentation of melanoma CTCs can be achieved. This fragmentation in turn would theoretically lead to the release of melanoma tumor antigens, with subsequent presentation to immune cells, which may help instigate an adaptive immune response.^38^^,^^39^ This hypothesis is particularly attractive in patients whose metastatic tumors are small or in locations that may not be amenable to other modalities that serve to augment treatment response to immunotherapy, such as radiation or the injection of cytolytic tumor vaccines. LA-PAT can also be applied to image CTCs in the lymph,^40^ but the weak optical absorption of lymph may require labeling in order to locate the lymphatic system. In addition to CTC detection, LA-PAT can measure other parameters, including depth, pH, oxygen saturation, stiffness, glucose metabolism, angiogenesis, and therapy response, to provide comprehensive information about solid tumors and metastasis.^41^^–^^46^

We have previously reported that LA-PAT is a potentially useful tool in the management of primary melanoma (*in vivo* determination of melanoma depth),^23^ and we now present data on its potential application in the detection and management of metastatic melanoma. We recognize that, due to the small number of patients imaged in this pilot study and the variable time of follow-up, the prognostic value of the detection of these circulating cells has not been determined. It is interesting, however, that the majority of patients without detected suspected CTCs had no disease progression over the short follow-up period. Larger controlled trials as described above, with extended clinical follow-up, will be necessary to determine optimal clinical applications.

The label-free and noninvasive nature of LA-PAT imaging of melanoma CTCs presents an inherent advantage for patients, avoiding the need for specific contrast agents or patient blood draws. LA-PAT could be an easily employable bedside modality for monitoring disease progression and response to therapy over time. Furthermore, our method relies on the production of melanin within melanoma cells, rather than the expression of cell surface receptors, and therefore offers an independent noninvasive modality that can complement other methods, such as *ex vivo* CTC detection assays that target cell surface receptors.^28^ Finally, the development of contrast agents for molecular photoacoustic imaging can extend the capability of LA-PAT to imaging cancer cells that do not express melanin.^47^ While future controlled studies with larger patient populations including *ex vivo* tumor cell validation are needed to definitively assess the prognostic value of LA-PAT detected CTCs, we believe that this is the first step toward the development of a technology that can constantly monitor CTCs in the circulatory system for disease management.
