# Supplementary material for: Label-free high-throughput photoacoustic tomography of suspected circulating melanoma tumor cells in patients in vivo
Source: J Biomed Opt. 2020 Mar 13;25(3):036002. doi: 10.1117/1.JBO.25.3.036002 (PMC7069252; doi:10.1117/1.JBO.25.3.036002)
Supplement: Supplementary file 1 [file JBO_025_036002_SD001.pdf]

Label-free High-throughput Photoacoustic Tomography of Suspected Circulating Melanoma Tumor Cells in Patients *in Vivo*

Pengfei Hai[1,2,†], Yuan Qu[1,†], Yang Li[1,2,†], Liren Zhu[1,2], Leonid Shmuylovich[3], Lynn A. Cornelius[3,*], and Lihong V. Wang[2,4,*]

[1]*Department of Biomedical Engineering, Washington University in St. Louis, St. Louis, MO 63130, USA*

[2]*Caltech Optical Imaging Laboratory, Andrew and Peggy Cherng Department of Medical Engineering, California Institute of Technology, Pasadena, CA 91125, USA*

[3]*Division of Dermatology, Washington University School of Medicine, St. Louis, MO 63110, USA*

[4]*Caltech Optical Imaging Laboratory, Department of Electrical Engineering, California Institute of Technology, Pasadena, CA 91125, USA*

Keywords: photoacoustic imaging, melanoma, circulating tumor cell.

† These authors contributed equally to this work.

* To whom correspondence should be addressed: L. A. C. (cornelil@wustl.edu) on melanoma and L. V. W. (lvw@caltech.edu) on PAT.

Supplementary Materials

Note S1: Detection sensitivity of melanoma CTC by LA-PAT

The ability to visualize a melanoma tumor cell in blood depends on the contrast-to-noise ratio (CNR), defined as

$$\mathrm{CNR} = \frac{\Delta\mathrm{PA}}{\sigma_{\mathrm{PA}}}. \tag{1}$$

Here, $\Delta\mathrm{PA}$ stands for the photoacoustic amplitude variation due to the strong optical absorption by the single melanoma CTC, and σ_{PA} stands for the standard deviation of the varying background photoacoustic amplitude due to the fluctuation in numbers of red blood cell (RBC).

The photoacoustic amplitude variation $\Delta\mathrm{PA}$ is defined as the photoacoustic amplitude difference between two adjacent voxels, with and without a melanoma CTC:

$$\Delta\mathrm{PA} = \mathrm{PA_w} - \mathrm{PA_{wo}}. \tag{2}$$

It can be further expressed as

$$\Delta\mathrm{PA} = \mathrm{PA_{CTC}} + \mathrm{PA_{RBC}}[\mathrm{RBC}]_\mathrm{w} - \mathrm{PA_{RBC}}[\mathrm{RBC}]_\mathrm{wo}. \tag{3}$$

Here $\mathrm{PA_{CTC}}$ stands for the photoacoustic signal from one melanoma CTC, and $\mathrm{PA_{RBC}}$ stands for the photoacoustic signal from one RBC. $[\mathrm{RBC}]_\mathrm{w}$ and $[\mathrm{RBC}]_\mathrm{wo}$stand for the numbers of RBCs in a resolution voxel, with and without a melanoma CTC.

Because the fluctuation of RBC number in each resolution voxel follows a Poisson distribution, the noise σ_{PA} can be expressed as

$$\sigma_{PA} = PA_{RBC} * \sqrt{[RBC]_{wo}}. \tag{4}$$

Combining equations (3) and (4), the CNR can be expressed as

$$CNR = \frac{PA_{CTC} + PA_{RBC} * [RBC]_w - PA_{RBC} * [RBC]_{wo}}{PA_{RBC} * \sqrt{[RBC]_{wo}}}. \tag{5}$$

It can be further simplified by canceling PA_{RBC}:

$$CNR = \frac{PA_{CTC}/PA_{RBC} + [RBC]_w - [RBC]_{wo}}{\sqrt{[RBC]_{wo}}}. \tag{6}$$

The photoacoustic amplitude ratio between one melanoma CTC and one RBC is determined by two factors, the cell volume and the absorption coefficient at the excitation wavelength. The volume ratio between a melanoma CTC and an RBC is typically ~23.3 (Assuming ~20 µm for the average diameter of melanoma CTCs, and ~7 µm for the average diameter of RBCs)[49,50]. By careful analysis of the absorption spectra of melanoma CTCs and RBCs in venous blood, the maximum absorption coefficient ratio is found to be ~64.5 (Fig. S1). Therefore, the typical value for PA_{CTC}/PA_{RBC} is ~1500. Because a melanoma CTC takes up only the space occupied by ~10 RBCs, the difference between $[RBC]_w$ and $[RBC]_{wo}$ is ~10, less than 1% of PA_{CTC}/PA_{RBC}, and can be neglected. So the CNR can be further expressed as

$$CNR = \frac{PA_{CTC}/PA_{RBC}}{\sqrt{[RBC]_{wo}}}. \tag{7}$$

$[RBC]_{wo}$ is determined by the number density of the RBCs in the blood and the resolution voxel size. The CNR, i.e., the melanoma CTC detection sensitivity, can be enhanced by reducing the resolution voxel size using an ultrasonic transducer with a higher frequency, at the expense of penetration depth. In this study, the spatial resolutions of the LA-PAT system were 43 µm in the

axial direction, 94 μm in the lateral direction, and 633 μm in the elevational direction. The resolution voxel size was ~2×10^{-3} μL. A typical RBC number density is ~5×10^{6} cells/μL, so $[\mathrm{RBC}]_{\mathrm{wo}}$ is ~1×10^{4} cells. Plugging in the values of $\mathrm{PA}_{\mathrm{CTC}}/\mathrm{PA}_{\mathrm{RBC}}$ and $[\mathrm{RBC}]_{\mathrm{wo}}$, the typical CNR of a single melanoma CTC is ~15, which is much higher than the threshold of 2 and indicates the high detection sensitivity of melanoma CTCs by LA-PAT.

It is worth noting that in the model above, noises from sources other than the fluctuation of RBCs are not considered because they are usually much smaller. However, as the imaging depth increases, the photoacoustic signals from RBCs attenuate and the noise σ_{PA} can be expressed as

$$\sigma_{\mathrm{PA}} = \mathrm{PA}_{\mathrm{RBC}} * \sqrt{[\mathrm{RBC}]_{\mathrm{wo}}} + \sigma_{\mathrm{o}}. \tag{8}$$

Here σ_{o} stands for noises due to sources other than the fluctuation of RBCs, mainly thermal acoustic noise from the medium, thermal noise from the ultrasonic transducer, and electronic noise from the amplifier. The CNR is then expressed as

$$\mathrm{CNR} = \frac{\mathrm{PA}_{\mathrm{CTC}}}{\mathrm{PA}_{\mathrm{RBC}} * \sqrt{[\mathrm{RBC}]_{\mathrm{wo}}} + \sigma_{\mathrm{o}}}. \tag{9}$$

The CNR decreases as the imaging depth increases, as was shown in Fig. 1.

Note S2: Estimation of melanoma CTC concentration in patients

The total number of melanoma CTCs N_t in a patient can be estimated by the following equation, assuming a random distribution of melanoma CTCs in blood:

$$N_t = N_d \frac{V_t}{V_f(t)}. \quad (10)$$

Here, N_d stands for the number of suspected melanoma CTCs detected by LA-PAT, V_t stands for the total blood volume in the patient, and $V_f(t)$ stands for the volume of blood imaged for the first time by LA-PAT in a given imaging time t.

To determine $V_f(t)$ that can sample during a given observation time t, we assume the following: (1) After each complete cycle of the blood stream we randomly sample a fraction γ of the entire blood volume, and (2) The blood is completely mixed at the end of each cycle. A cycle is defined as the time it takes to pump the entire blood volume through the body:

$$T = \frac{V_t}{Q_{CO}}. \quad (11)$$

Here, Q_{CO} stands for the average cardiac output (as a volumetric flow rate, or volume of blood per unit time) defined as

$$Q_{CO} = r_{HR} \times V_{SV}. \quad (12)$$

Further, r_{HR} stands for the heart rate of the patient and V_{SV} represents the stroke volume. So the cycle duration becomes

$$T = \frac{V_t}{r_{HR} \times V_{SV}}. \quad (13)$$

The sampling ratio γ of the entire blood stream by the observed vein is defined as

$$\gamma = \frac{Q_O}{Q_{CO}} = \frac{Q_O T}{V_t}. \tag{14}$$

Here, Q_O stands for the volumetric flow rate of the blood observed by LA-PAT (the subscript "O" stands for "observed"). Because we want to capture the circulating melanoma CTC multiple times, Q_O is always limited by the volumetric flow rate of the vein monitored and can be expressed as

$$Q_O = \pi \times \frac{D^2}{4} \times v. \tag{15}$$

Here, D stands for the diameter of the vein and v stands for the average blood flow speed.

During a given observation time t, if $t \le T$, in which case there is no blood sampled repeatedly by LA-PAT, the volume of blood imaged for the first time can be calculated by the following equation:

$$V_f(t) = Q_O \times t. \tag{16}$$

If $t > T$, in which case multiple cycles occur during the observation time, at cycle i, the volume of blood imaged for the first time is

$$V_i = Q_O T (1-\gamma)^{i-1}. \tag{17}$$

Therefore, the total volume of blood imaged for the first time during the observation time t is

$$\begin{aligned} V_f(t) &= \sum_{i=1}^{\left\lfloor \frac{t}{T} \right\rfloor} Q_O T (1-\gamma)^{i-1} + Q_O \left(t - \left\lfloor \frac{t}{T} \right\rfloor T\right)(1-\gamma)^{\left\lfloor \frac{t}{T} \right\rfloor} \\ &= Q_O T \frac{1-(1-\gamma)^{\left\lfloor \frac{t}{T} \right\rfloor}}{\gamma} + Q_O \left(t - \left\lfloor \frac{t}{T} \right\rfloor T\right)(1-\gamma)^{\left\lfloor \frac{t}{T} \right\rfloor} \end{aligned} \tag{18}$$

$$= V_t[1 - (1 - \frac{Q_O T}{V_t})^{\lfloor \frac{t}{T} \rfloor}] + Q_O\left(t - \left\lfloor \frac{t}{T} \right\rfloor T\right)(1 - \frac{Q_O T}{V_t})^{\lfloor \frac{t}{T} \rfloor}.$$

Here, $\lfloor a \rfloor$ is the largest integer that is less than or equal to the real number a. Briefly, the expression consists of two parts: the sum of a geometric series representing a decreasingly efficient sampling of the blood stream at each full cycle, and a residual term at the last incomplete cycle. If we had infinite time, mathematically, we could ignore the residual term and take the infinite limit of the sum:

$$\lim_{t \to +\infty} V_f(t) = \lim_{N \to +\infty} V_t[1 - (1 - \frac{Q_O T}{V_t})^N] = V_t. \qquad (19)$$

Therefore, the entire blood volume can be sampled. In summary, the total number of melanoma CTCs N_t can be estimated by

$$N_t = N_d \frac{V_t}{V_f(t)}$$

$$= \begin{cases} N_d \dfrac{V_t}{Q_O t} & \text{if } t < T \\ N_d \dfrac{V_t}{V_t(1 - (1 - \frac{Q_O T}{V_t})^{\lfloor \frac{t}{T} \rfloor}) + Q_O\left(t - \left\lfloor \frac{t}{T} \right\rfloor T\right)(1 - \frac{Q_O T}{V_t})^{\lfloor \frac{t}{T} \rfloor}} & \text{if } t > T \end{cases} \qquad (20)$$

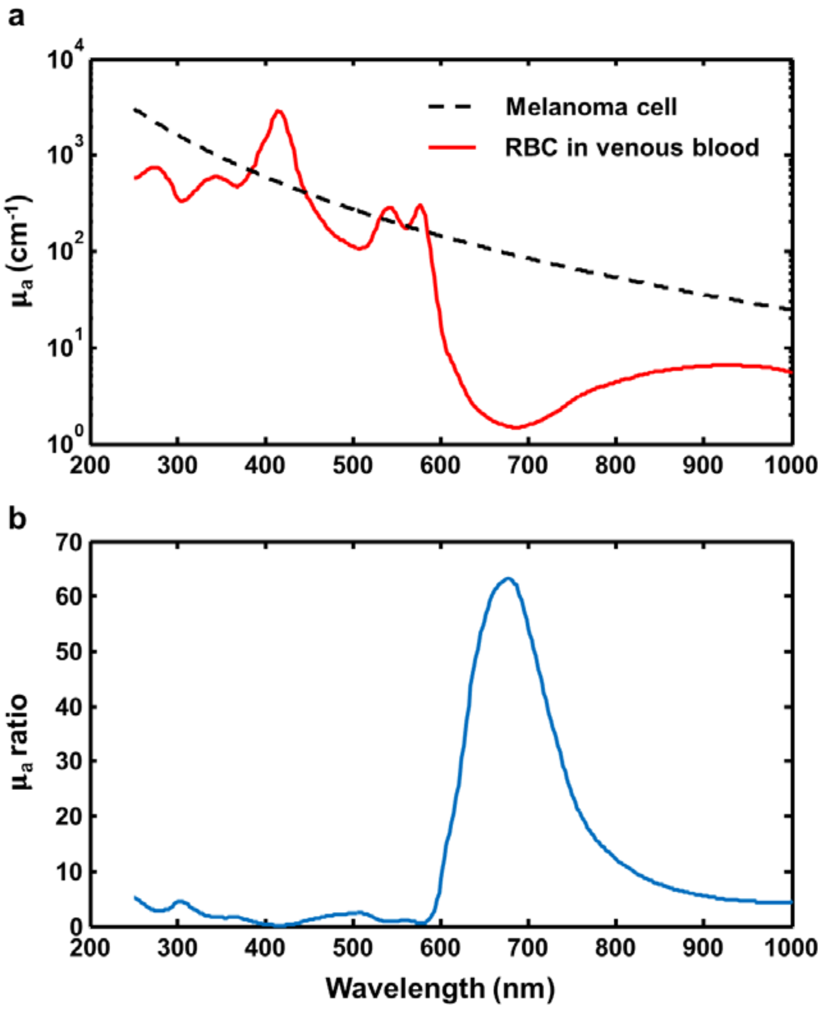

Fig S1: Optimal excitation wavelength for photoacoustic imaging of melanoma CTCs. (**A**) Optical absorption spectra of melanoma cells (assuming an average of 43% volume fraction of melanosome; the average absorption coefficient for a melanosome was referenced from the literature)[48] and RBCs in venous blood (84% ±2.7% oxygen saturation)[19]. μ_a, optical absorption coefficient. The number of RBCs in a resolution voxel is in between 1.0×10^4 and 1.5×10^4. (**B**) absorption coefficient ratio of melanoma cells to RBCs in venous blood. An excitation wavelength of 680 nm was chosen to maximize the melanoma CTC detection sensitivity.

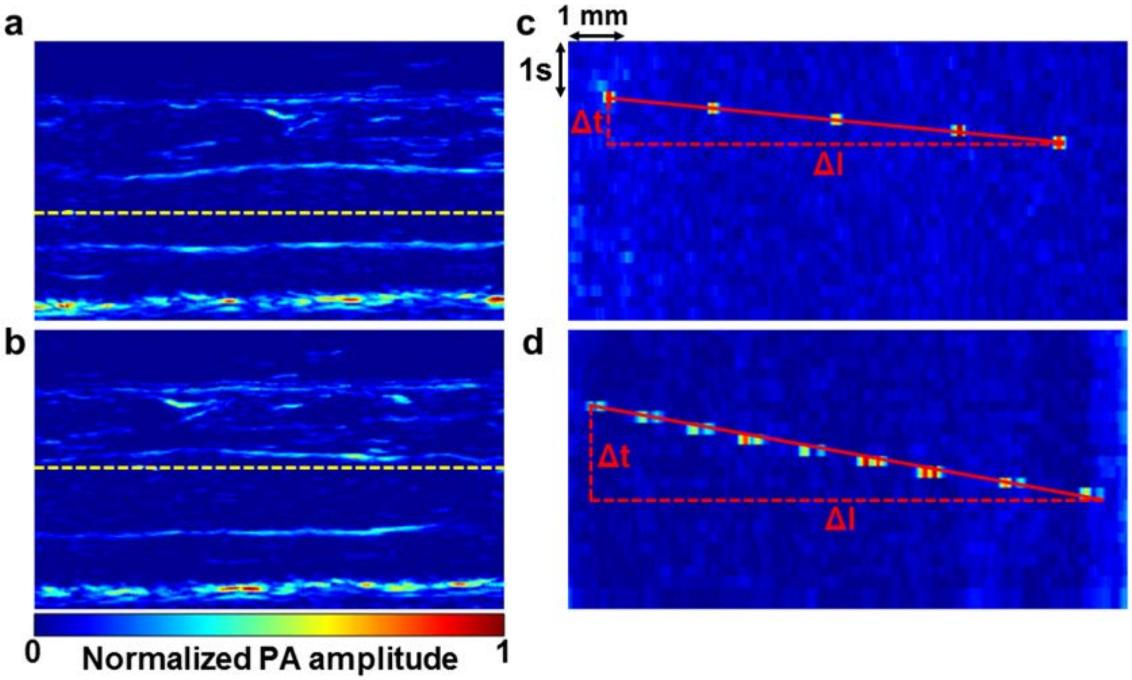

Fig S2. Estimation of suspected melanoma CTC flow speed. (**A**) A representative photoacoustic image of a single melanoma CTC in a patient. (**B**) A representative photoacoustic image of a melanoma CTC cluster in the patient. (**C**) The time trace plot of each pixel along the yellow dashed line in (**A**) in the space-time domain. The slope of the red solid line, computed by linear fitting as 10.3 mm/s, represents the flow speed of the single melanoma CTC. (**D**) The time trace plot of each pixel along the yellow dashed line in (**B**) in the space-time domain. The slope of the red solid line, computed by linear fitting as 5.4 mm/s, represents the flow speed of the melanoma CTC cluster. This method cannot measure the flow speed of blood stream directly, because it needs to track a discrete PA signal source.

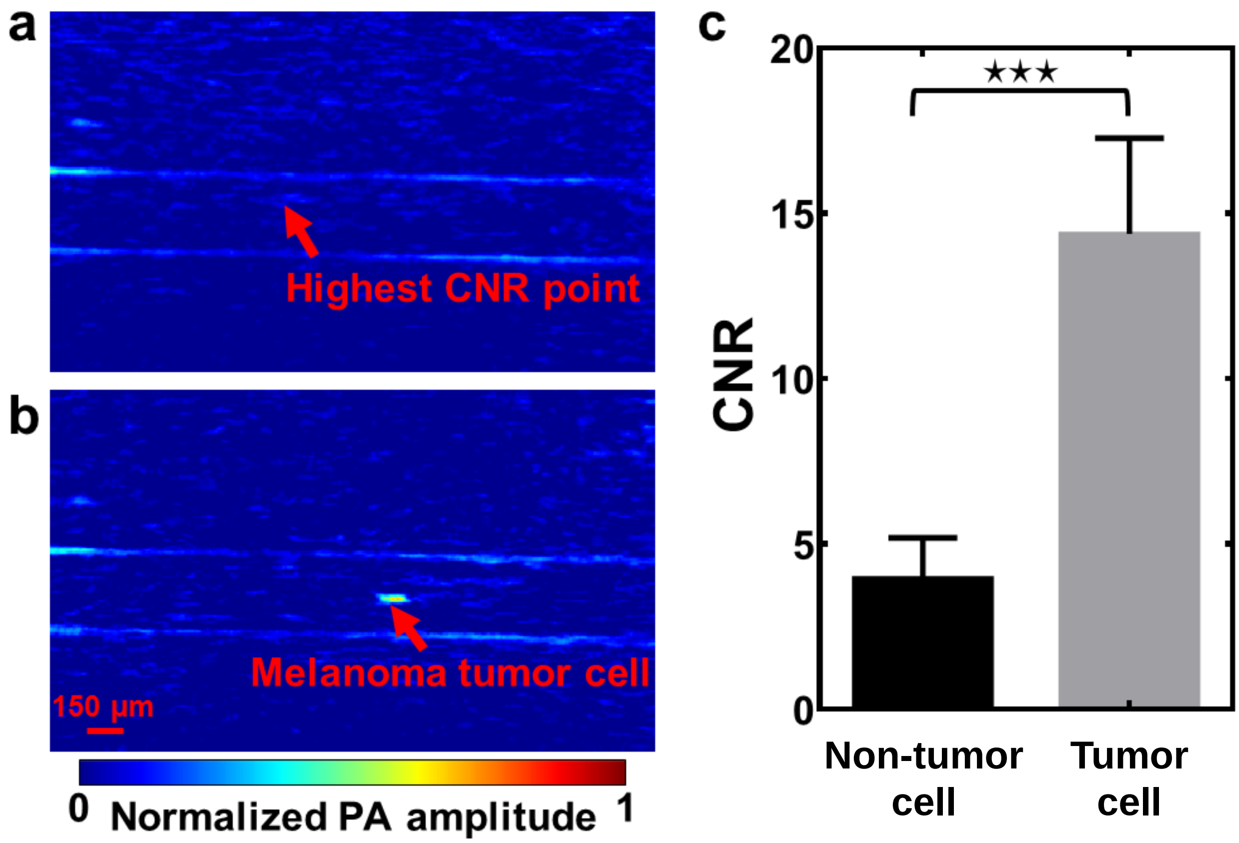

Fig S3. The CNRs of melanoma tumor cells were much higher than the peak CNRs in the microtube in phantom. (**A**) A representative photoacoustic image of the phantom without melanoma tumor cells. (**B**) A representative photoacoustic image of the phantom with a melanoma tumor cell. The flowing object with the highest CNR was identified as a melanoma tumor cell. (**C**) CNRs of melanoma tumor cells were much higher than peak CNRs in the microtube in phantom (n = 105 melanoma tumor cell events, ***$p < 0.001$).

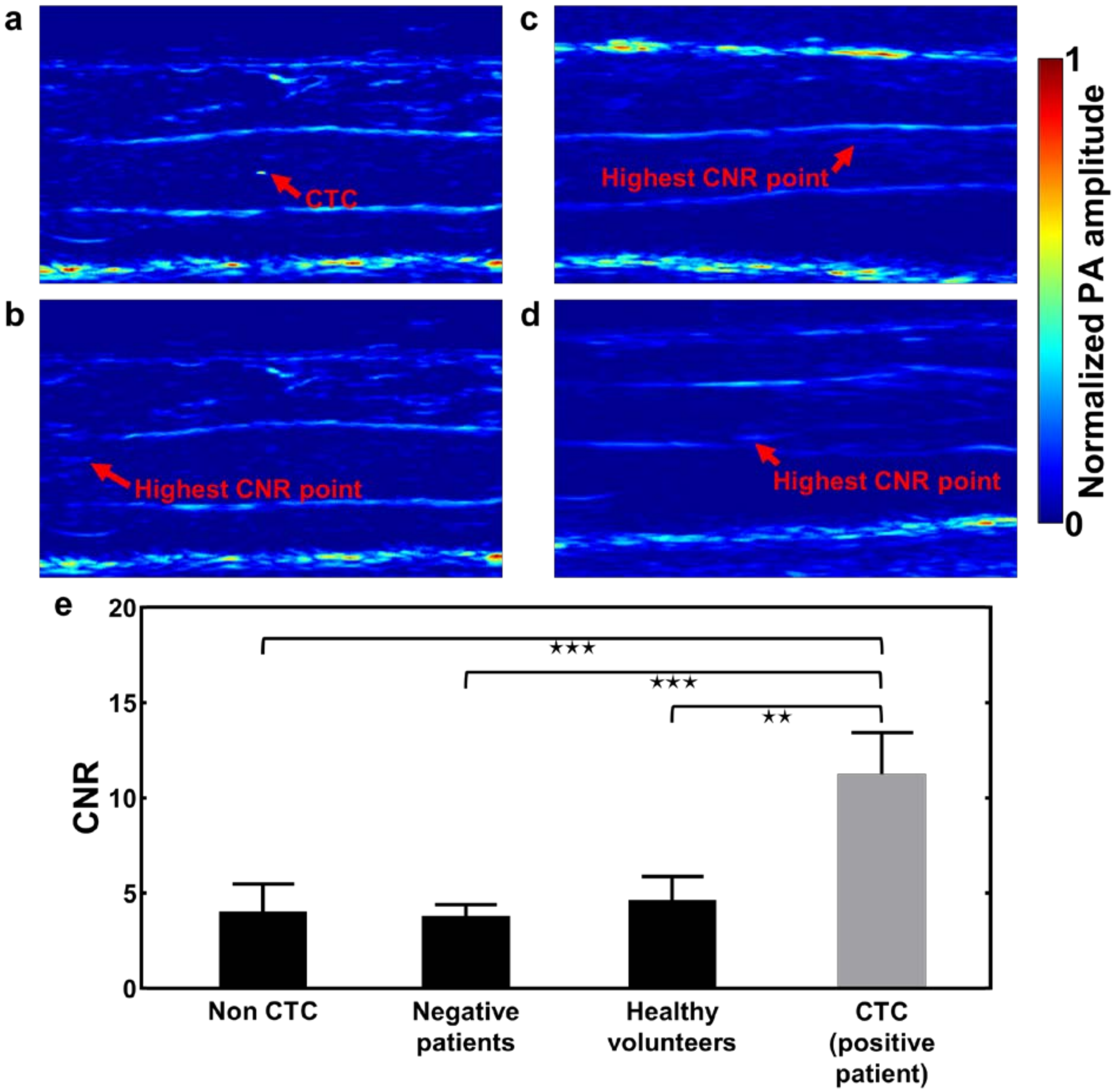

Fig S4. The CNRs of suspected melanoma CTCs were much higher than the peak CNRs in blood vessels. (**A**) A representative photoacoustic image of a melanoma CTC in a blood vessel in a patient, referred to as a positive patient. The circulating object with the highest CNR was identified as a melanoma CTC. (**B**) A representative photoacoustic image without melanoma CTCs from the positive patient. (**C**) A representative photoacoustic image from a patient in whom no melanoma CTCs were detected, referred to as a negative patient. (**D**) A representative

photoacoustic image from a healthy volunteer. (**E**) CNRs of melanoma CTCs were much higher than peak CNRs in blood vessels in positive patients, negative patients and healthy volunteers ($n = 4$ melanoma CTC events detected in 3 patients, measured across 22 video frames, ***$p < 0.001$, **$p < 0.01$).

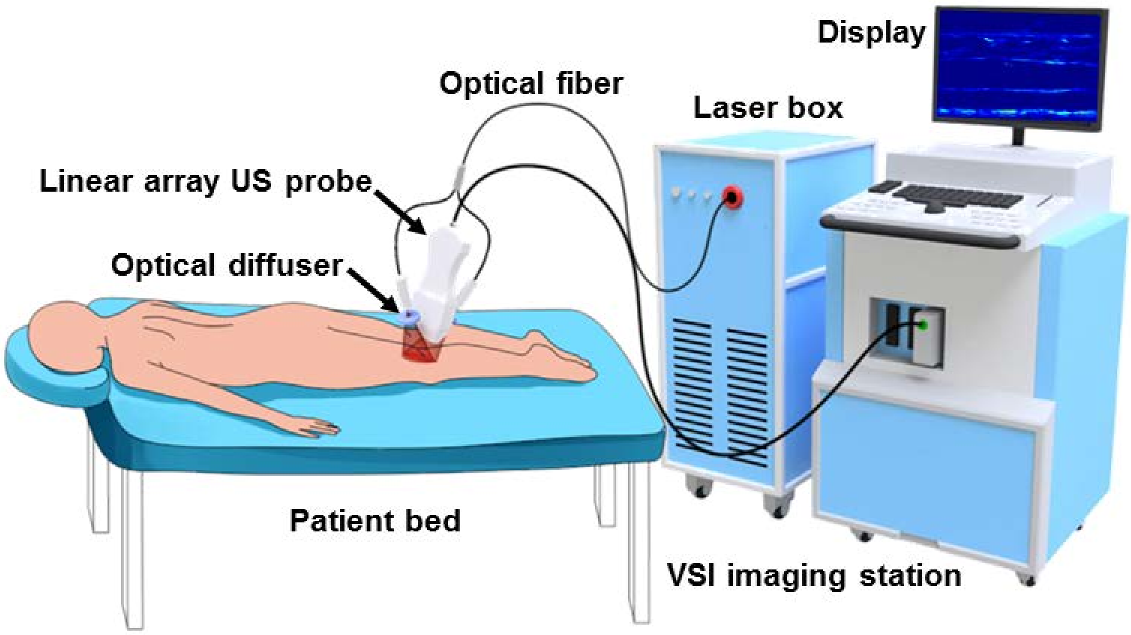

Fig S5. System schematic of LA-PAT for melanoma CTC imaging in patients.

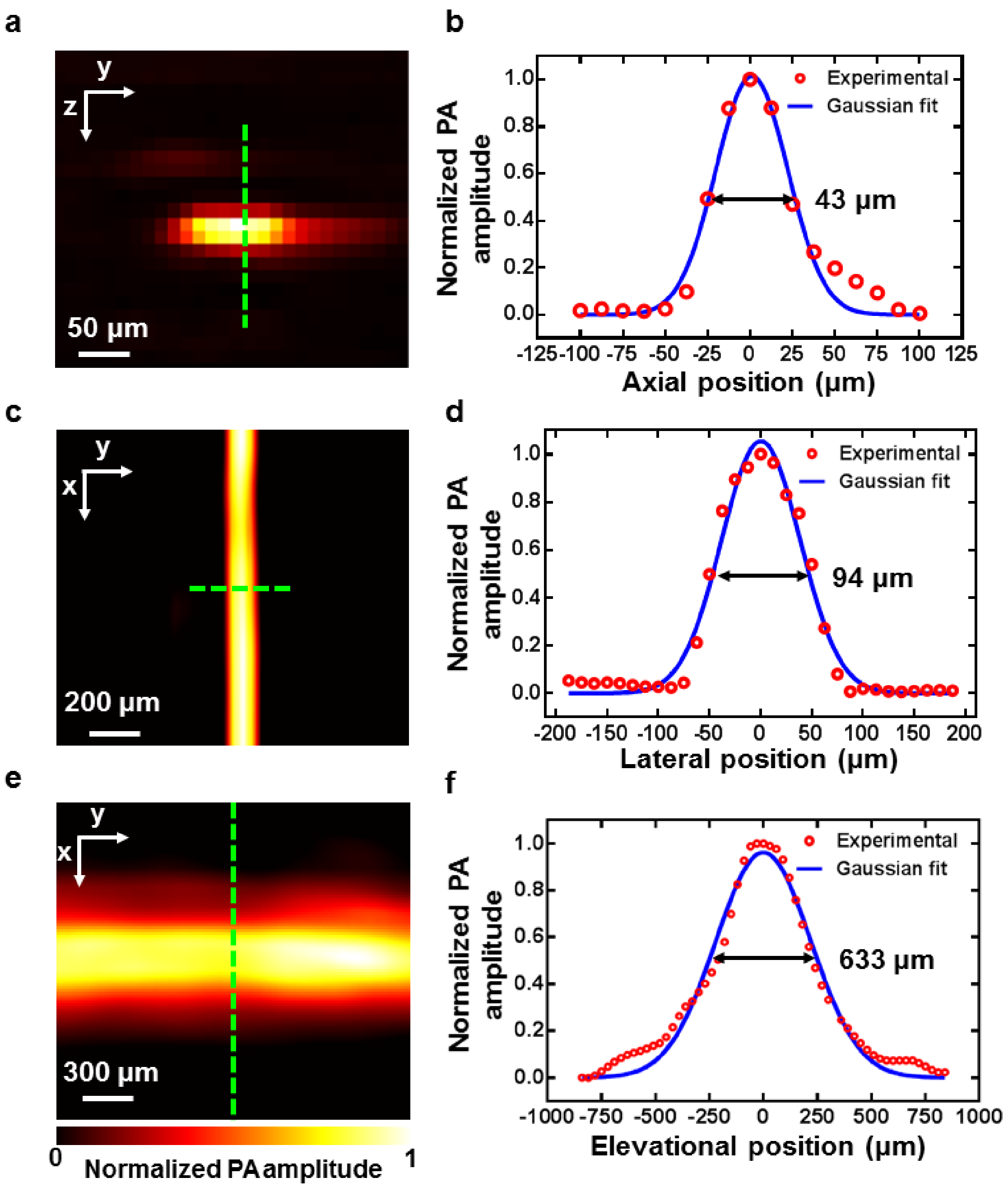

Fig S6. Quantification of spatial resolutions of LA-PAT system. (**A**) A cross-sectional image of a carbon fiber by LA-PAT. (**B**) Photoacoustic amplitude profile along the green dashed line in (**A**). The profile was fitted to a Gaussian function, and the full width at half maximum (FWHM) was quantified to be 43 μm as the axial resolution of the LA-PAT system. (**C**) A maximum amplitude projection (MAP) image along the axial direction of the carbon fiber by LA-PAT. (**D**)

The photoacoustic amplitude profile along the green dashed line in (**C**). The profile was fitted to a Gaussian function and the FWHM was quantified to be 94 μm as the lateral resolution of the LA-PAT system. (**E**) A MAP image along the axial direction of the carbon fiber by LA-PAT. (**F**) The photoacoustic amplitude profile along the green dashed line in (**E**). The profile was fitted to a Gaussian function and the FWHM was quantified to be 633 μm as the elevational resolution of the LA-PAT system.

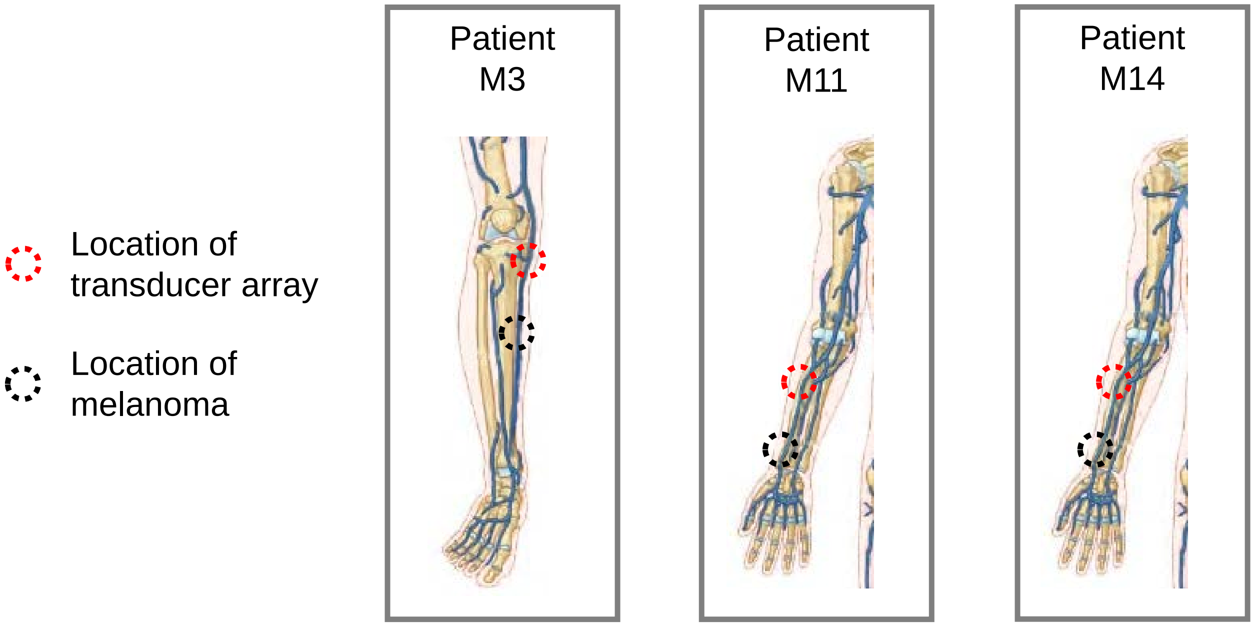

Fig S7. Schematic showing the melanoma and ultrasonic transducer array locations.

Table S1: Status of CTC-positive patients

**Patient ID**	M3	M11	M14
**Clinical stage at time of scan**	IIIB	IV	IIIC
**CTCs imaged by LA-PAT**	3-5	1	2-4
**Effective blood volume examined**	46.8 ml	15.0 ml	13.5 ml
**Estimated total CTC count per milliliter**	0.06-0.11	0.07	0.15-0.30
**Clinical status at time of LA-PAT scan**	Loco-regional disease	Visceral disease	Local recurrence
**Follow-up clinical status**	Disease progression	Disease progression	No disease progression
**Time of follow-up post LA-PAT scan**	19 months	14 months	13 months

Table S2: List of melanoma patients imaged by LA-PAT

Patient ID	LA-PAT Imaging	Clinical stage at time of scan	Follow-up clinical status	Time of follow up post LA-PAT	Sampled volume (ml)
M1	Negative	IV	Disease progression	13 months	40.1
M2	Negative	IIIC	No disease progression	4 months	1.7
M3	Positive	IIIB	Disease progression	19 months	46.8
M4	Negative	IIIA	No disease progression	18 months	12.0
M5	Negative	IV	No disease progression	15 months	8.0
M6	Negative	IIIC	Disease progression	15 months	7.0
M7	Negative	IV	No disease progression	17 months	8.5
M8	Negative	IV	Disease progression	8 months	5.4
M9	Negative	IIIC	No disease progression	15 months	9.0
M10	Negative	IV	No disease progression	15 months	2.8
M11	Positive	IV	Disease progression	14 months	15.0
M12	Negative	IV	No disease progression	14 months	1.5
M13	Negative	IIIC	No disease progression	15 months	6.8
M14	Positive	IIIC	No disease progression	13 months	13.5
M15	Negative	IV	No disease progression	12 months	33.4
M16	Negative	IV	Disease progression	15 months	5.5
